# Supplementary material for: Identification of Freezing-Responsive microRNAs and Their Targets in Chinese Jujube by Small RNA and Degradome Sequencing
Source: Int J Mol Sci. 2026 Sep 4;27(17):7912. doi: 10.3390/ijms27177912 (PMC13566890; doi:10.3390/ijms27177912)
Supplement: Supplementary file 1 [file ijms-27-07912-s001.zip › Supplementary Material S3.pdf]

Table S3. miRNA Family annotation

miRNA	Family
novel_miR_207	bantam
novel_miR_220	MIR1428
gra-miR157b	MIR156
mes-miR156i	MIR156
fve-miR156g-5p	MIR156
vvi-miR156g	MIR156
mtr-miR156b-3p	MIR156
cpa-miR156e	MIR156
rco-miR156g	MIR156
lus-miR156h	MIR156
gra-miR157a	MIR156
vvi-miR156i	MIR156
tcc-miR156e	MIR156
bgy-miR156	MIR156
ptc-miR156j	MIR156
ptc-miR156g	MIR156
ppe-miR156g	MIR156
fve-miR156g-3p	MIR156
mes-miR156j	MIR156
ptc-miR156i	MIR156
bcy-miR156	MIR156
mes-miR156h	MIR156
rco-miR156h	MIR156
novel_miR_137	MIR158
novel_miR_71	MIR158
novel_miR_148	MIR158
novel_miR_22	MIR158
novel_miR_178	MIR158
ctr-miR319	MIR159
ptc-miR319a	MIR159
csi-miR159c-3p	MIR159
rco-miR319a	MIR159
gma-miR319m	MIR159
vvi-miR319f	MIR159
mdm-miR319d	MIR159
vvi-miR319c	MIR159
nta-miR319b	MIR159
vun-miR319a	MIR159
hbr-miR319	MIR159
hbr-miR159a	MIR159
cpa-miR160c-5p	MIR160

fve-miR160b	MIR160
ptc-miR160b-5p	MIR160
vvi-miR160e	MIR160
mes-miR160d	MIR160
ptc-miR160c-5p	MIR160
mdm-miR160e	MIR160
mes-miR160h	MIR160
cpa-miR160f-5p	MIR160
eun-miR162-5p	MIR162_1
fve-miR162-5p	MIR162_1
eun-miR162-3p	MIR162_1
vun-miR162	MIR162_2
mdm-miR162b	MIR162_2
gma-miR162c	MIR162_2
mes-miR162	MIR162_2
mdm-miR162a	MIR162_2
fve-miR162-3p	MIR162_2
novel_miR_88	MIR164
novel_miR_198	MIR164
novel_miR_122	MIR164
novel_miR_152	MIR164
novel_miR_27	MIR164
lus-miR166d	MIR166
ptc-miR166h	MIR166
vvi-miR166h	MIR166
tcc-miR166d	MIR166
novel_miR_48	MIR166
ppe-miR166a	MIR166
mdm-miR166i	MIR166
mdm-miR166e	MIR166
rco-miR167c	MIR167_1
cme-miR167c	MIR167_1
mes-miR167b	MIR167_1
csi-miR167b-5p	MIR167_1
tcc-miR167a	MIR167_1
csi-miR167b-3p	MIR167_1
lja-miR167	MIR167_1
rco-miR171a	MIR171_1
mtr-miR171e-5p	MIR171_1
gma-miR171f	MIR171_1
gma-miR171l	MIR171_1
lja-miR171d-3p	MIR171_1
fve-miR171g	MIR171_1
fve-miR171f-5p	MIR171_1

ppe-miR171g	MIR171_1
csi-miR171a	MIR171_1
ppe-miR171a	MIR171_1
gma-miR171k-5p	MIR171_1
mdm-miR171f-3p	MIR171_1
cme-miR171b	MIR171_1
novel_miR_206	MIR171_2
novel_miR_196	MIR171_2
novel_miR_142	MIR171_2
novel_miR_211	MIR171_2
novel_miR_170	MIR171_2
novel_miR_57	MIR171_2
novel_miR_182	MIR171_2
novel_miR_131	MIR171_2
novel_miR_60	MIR172
novel_miR_228	MIR172
novel_miR_226	MIR172
novel_miR_159	MIR1862
novel_miR_165	MIR1878
novel_miR_110	MIR1886
novel_miR_103	MIR2108
ptc-miR2111b	MIR2111
fve-miR2111b-5p	MIR2111
novel_miR_139	MIR2118
novel_miR_194	MIR2863
novel_miR_181	MIR2950
novel_miR_19	MIR319
mtr-miR319b-3p	MIR319
gma-miR319a	MIR319
ptc-miR319c	MIR319
novel_miR_2	MIR3434
novel_miR_171	MIR3434
novel_miR_176	MIR3434
novel_miR_46	MIR390
novel_miR_105	MIR390
ptc-miR393a-5p	MIR393
ptc-miR393b-5p	MIR393
ptc-miR393a-3p	MIR393
vvi-miR393b	MIR393
ptc-miR393b-3p	MIR393
vvi-miR395d	MIR395
bra-miR395a-3p	MIR395
cas-miR395a	MIR395
bna-miR395a	MIR395

novel_miR_174	MIR396
novel_miR_74	MIR396
novel_miR_188	MIR396
novel_miR_21	MIR396
novel_miR_52	MIR396
novel_miR_14	MIR396
novel_miR_147	MIR398
novel_miR_214	MIR398
novel_miR_146	MIR399
novel_miR_79	MIR403
novel_miR_62	MIR403
novel_miR_121	MIR403
novel_miR_8	MIR408
novel_miR_12	MIR4221
novel_miR_96	MIR4368
csi-miR156j-5p	MIR4414
novel_miR_133	MIR474
novel_miR_129	MIR477
novel_miR_32	MIR477
mdm-miR482a-5p	MIR482
novel_miR_101	MIR482
novel_miR_1	MIR482
novel_miR_63	MIR482
novel_miR_77	MIR482
novel_miR_219	MIR482
novel_miR_82	MIR482
novel_miR_113	MIR482
novel_miR_108	MIR482
mdm-miR482a-3p	MIR482
novel_miR_59	MIR482
novel_miR_6	MIR482
novel_miR_200	MIR482
novel_miR_3	MIR5270
osa-miR5523	MIR5270
novel_miR_168	MIR5291
novel_miR_118	MIR530
novel_miR_5	MIR5380
novel_miR_128	MIR5565
novel_miR_26	MIR5568
novel_miR_39	MIR5568
novel_miR_172	MIR5568
novel_miR_192	MIR5630
novel_miR_150	MIR5752
novel_miR_78	MIR6019

novel_miR_112	MIR6267
novel_miR_222	MIR6426
novel_miR_66	MIR7492
novel_miR_84	MIR7492
novel_miR_93	MIR7492
novel_miR_201	MIR7502
novel_miR_120	MIR7729
novel_miR_117	MIR7729
novel_miR_81	MIR7729
novel_miR_169	MIR7729
novel_miR_68	MIR7757
novel_miR_204	MIR781
novel_miR_155	MIR862_2
novel_miR_158	MIR8674
novel_miR_153	MIR902
gma-miR5368	MIR9408
novel_miR_177	MIR9408
novel_miR_25	MIR9483
novel_miR_83	MIR9560
novel_miR_106	MIR9674
